# Retraction Note: Targeting a novel LncRNA SNHG15/miR-451/c-Myc signaling cascade is effective to hamper the pathogenesis of breast cancer (BC) in vitro and in vivo

**DOI:** 10.1186/s12935-023-03049-8

**Published:** 2023-09-14

**Authors:** Jiang Du, Hong Zhong, Binlin Ma

**Affiliations:** Department of Breast and Thyroid Surgery, The 3rd Affiliated Teaching Hospital of Xinjiang Medical University (Affiliated Cancer Hospital), Suzhou East Street No. 789, Xinshi District, Urumqi, 830011 Xinjiang China

**Retraction Note: Cancer Cell International (2021) 21:186**

10.1186/s12935-021-01885-0

The Editors in Chief have retracted this article. After publication, concerns were raised about multiple images that appear to overlap with previously-published works, namely:

Figure 6a overlaps with Figure 9C from [1].

Figure 6a overlaps with Figure 2I from [2].

Figure 6a overlaps with Figure 8A from [3].

Figure 5c and d overlap with Figure 1R from [4].

Figure 6c overlaps with Figure 5G from [5].

Figures 3g and 4g overlap with Figures 4L, 4M, 6L and 6M from [6].

Supplementary Figure S2A overlaps with Figure 4C from [7].

Supplementary Figure S2A overlaps with Figure 5A from [8].

The authors have stated that they had used third-party services that provided them with data, Therefore, the Editors in Chief have lost confidence in the findings of this paper. All authors agree to this retraction.
